# A Roving Dual-Presentation Simultaneity-Judgment Task to Estimate the Point of Subjective Simultaneity

**DOI:** 10.3389/fpsyg.2016.00416

**Published:** 2016-03-24

**Authors:** Kielan Yarrow, Sian E. Martin, Steven Di Costa, Joshua A. Solomon, Derek H. Arnold

**Affiliations:** ^1^Department of Psychology, City University LondonLondon, UK; ^2^Department of Psychology, UCL Institute of Cognitive NeuroscienceLondon, UK; ^3^Centre for Applied Vision Science, City University LondonLondon, UK; ^4^School of Psychology, The University of QueenslandBrisbane, QLD, Australia

**Keywords:** multisensory perception, timing and time perception, temporal order, 2AFC, simultaneity judgment

## Abstract

The most popular tasks with which to investigate the perception of subjective synchrony are the temporal order judgment (TOJ) and the simultaneity judgment (SJ). Here, we discuss a complementary approach—a dual-presentation (2x) SJ task—and focus on appropriate analysis methods for a theoretically desirable “roving” design. Two stimulus pairs are presented on each trial and the observer must select the most synchronous. To demonstrate this approach, in Experiment 1 we tested the 2xSJ task alongside TOJ, SJ, and simple reaction-time (RT) tasks using audiovisual stimuli. We interpret responses from each task using detection-theoretic models, which assume variable arrival times for sensory signals at critical brain structures for timing perception. All tasks provide similar estimates of the point of subjective simultaneity (PSS) on average, and PSS estimates from some tasks were correlated on an individual basis. The 2xSJ task produced lower and more stable estimates of model-based (and thus comparable) sensory/decision noise than the TOJ. In Experiment 2 we obtained similar results using RT, TOJ, ternary, and 2xSJ tasks for all combinations of auditory, visual, and tactile stimuli. In Experiment 3 we investigated attentional prior entry, using both TOJs and 2xSJs. We found that estimates of prior-entry magnitude correlated across these tasks. Overall, our study establishes the practicality of the roving dual-presentation SJ task, but also illustrates the additional complexity of the procedure. We consider ways in which this task might complement more traditional procedures, particularly when it is important to estimate both PSS and sensory/decisional noise.

## Introduction

Introspection suggests conscious experiences proceed successively. This is part of what we mean when we say that we have a sensation of the passage of time. Determining the relative timing at which two or more events occur would thus appear to be an important perceptual operation, and one that might underscore various higher-level inferences, such as the causal relationship between events, or the degree to which two events should be grouped perceptually. However, the processes by which the brain determines relative timing require clarification. The problem appears particularly acute for multisensory events, where relevant neural signals might be dispersed widely in space and time. However, even within a single sense, the way in which temporal succession and overlap are determined is not yet established.

One of the most fundamental questions one can ask about relative timing is with what objective asynchrony the observer considers two events to be maximally synchronous. We usually investigate this issue by attempting to estimate a point of subjective simultaneity (PSS) from different experimental conditions. Hence, to make progress, we need good experimental procedures to lay bare our temporal qualia. In this paper we consider a complementary task for this purpose: The dual-presentation simultaneity judgment (2xSJ). This type of task has been used fairly infrequently in relative timing experiments (e.g., Allan and Kristofferson, 1974; Van de Par and Kohlrausch, 2000; Powers et al., 2009; Roseboom et al., 2011; Stevenson and Wallace, 2013). Here we will first argue that a roving-standard design is theoretically desirable, second describe an appropriate observer model for fitting and summarizing the data this task generates, and third attempt to benchmark data from this task against more common approaches in order to assess its strengths and limitations.

## Temporal judgment tasks

**Figure 1:**
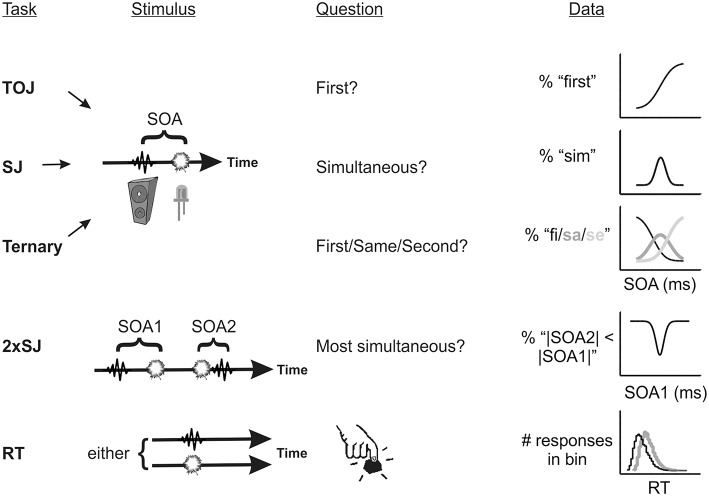
**Schematic of the five tasks used in Experiments 1–3, incorporating the stimulus timeline and predicted psychometric functions (or response histograms)**. TOJ, temporal order judgment; SJ, simultaneity judgment; 2xSJ, dual-presentation SJ; RT, simple reaction time.

For explicit temporal judgments, two tasks are particularly popular: The temporal order judgment task (TOJ; e.g., Sternberg and Knoll, 1973) and the synchrony judgment task (SJ; e.g., Schneider and Bavelier, 2003). The former task asks of a participant “which came first” (or some variant) whereas the latter asks “were they simultaneous?” Another somewhat less utilized variant, the ternary order or SJ3 task (e.g., Ulrich, 1987), offers three response categories: “first,” “simultaneous,” and “second.” In all cases trial-by-trial data can be summarized via meaningful model parameters when an appropriate observer model is fitted. These tasks (and two further tasks which will be discussed shortly) are schematized in Figure 1. The most commonly derived parameter, the point of subjective simultaneity (or PSS), captures any bias to report one stimulus as having come earlier than the other.

For the temporal order judgment task, under typical observer models (e.g., Gibbon and Rutschmann, 1969) the PSS estimate can be inferred to represent a combination of, first, a difference in sensory delays for the two signals and, second, any decision-level bias in interpreting relative arrival times. Distinguishing these contributions is not generally possible, which raises interpretative issues, particularly if decision-level biases might reasonably be expected to change across experimental conditions. For example, “prior entry” (Titchener, 1908) describes an experimental finding wherein attended events are thought to be perceived more rapidly than unattended ones (see Spence and Parise, 2010, for review). Prior entry has often been assessed using TOJs, with two stimuli originating from different positions and/or sensory modalities, and attention directed preferentially toward one of the two events. The demand characteristic, to attend preferentially to one of the two stimulus origins, has the potential to place that particular answer firmly in mind, which might bias responses at the decision level (Shore et al., 2001; Spence et al., 2001).

The simultaneity judgment and ternary order judgment tasks can also be used to recover a PSS, and in some cases, such as the prior entry effect, these tasks might be more appropriate in order to make the question less leading.^1^ However, rather than a PSS, these tasks most naturally recover two boundaries around subjective simultaneity, reflecting points where judgments change from “A precedes B” to “simultaneous,” and from “simultaneous” to “A follows B” (Yarrow et al., 2011). Hence it is quite common to observe a plateau in the psychometric function, with simultaneity reported ubiquitously across several SOAs (for some examples, see García-Pérez and Alcalá-Quintana, 2012a, **Figures 6–8**; Yarrow et al., 2015, **Figure 4**). Inferring a single PSS from such data requires additional assumptions (e.g., whether the threshold for perceiving/judging simultaneity is the same when A follows B as when B follows A) which may be problematic, as there is no current consensus regarding the correct observer model for data in this form (e.g., Ulrich, 1987; Schneider and Bavelier, 2003; Yarrow et al., 2011; García-Pérez and Alcalá-Quintana, 2012a,b). In a sense, SJ and SJ3 tasks provide a temporal window within which the PSS lies, rather than a single point estimate.

## Observer models for characterizing temporal judgements

So far we have made reference to observer models without specifying exactly what this means. In this paper we will use observer models derived from signal detection theory (SDT; Green and Swets, 1966; Macmillan and Creelman, 2005). Detection-theoretic approaches to temporal judgments are well-established (e.g., Baron, 1969; Gibbon and Rutschmann, 1969; Sternberg and Knoll, 1973; Allan, 1975; Ulrich, 1987; Schneider and Bavelier, 2003; Yarrow et al., 2011, 2013; García-Pérez and Alcalá-Quintana, 2012a,b). Models of this type generally assume that observers are accessing a (noisy) encoding of the *difference in arrival times* (Δ*t*) between two signals (somewhere in the brain) and using this quantity to make a decision. The key source of noise in these decisions is variability in terms of the latency with which signals arrive at a decisional mechanism, with each signal contributing its (additive) variability to the distribution of encoded differences in arrival times across trials. This kind of model has been referred to as a general independent-channels model (Sternberg and Knoll, 1973) or a general-threshold model (Ulrich, 1987).

Specific variants of this general model vary mostly in terms of how many additional layers of complexity are included. For example, the simplest way to conceive of a temporal order judgment (TOJ) is that there is a single criterion used to divide the observed Δ*t* into two possible order responses (Gibbon and Rutschmann, 1969). If the difference in arrival times falls below this criterion, event A is judged as having happened first; otherwise it is judged second. If, during an experiment, two stimuli are presented repeatedly but at varying physical stimulus onset asynchronies (SOAs), the model predicts a smooth function relating the SOA to the proportion of times one of the two orders is selected. The shape of this function reflects the form of latency noise, being, for example, a cumulative Gaussian if Gaussian latency noise is assumed (Baron, 1969).

Variants of these models can make predictions about other common temporal judgments in addition to the TOJ, such as the SJ3 task (before/same/after), considered in detail by Allan (1975) and Ulrich (1987), and the SJ task, considered for example by Schneider and Bavelier (2003) and by Yarrow et al. (2011, 2013). In these tasks the internal response Δ*t* must be divided into three regions, rather than two (in order to demarcate “same” from “before” and “after”). This means there are two decision criteria, not one. In a variant of this kind of model, some authors (e.g., Venables, 1960; García-Pérez and Alcalá-Quintana, 2012a,b) consider that there might also be a zone near zero where no differentiation of timing is possible and observers must guess. This inclusion, of a “guessing zone,” is a departure from classic SDT, which avoids the notion of a hard threshold. Instead, classic SDT presumes that encoded values are always recoverable. Another feature that can vary between models is the form of assumed latency noise (for example, exponential rather than Gaussian arrival time distributions can be assumed; García-Pérez and Alcalá-Quintana, 2012a,b).

## A roving dual-presentation SJ task

In this paper we consider a variant of the popular SJ task, which we refer to as a (roving) 2xSJ task. This task has a close structural similarity to some recent approaches in visual psychophysics (Morgan et al., 2013, 2015; Jogan and Stocker, 2014; García-Pérez and Peli, 2014). Although roving 2xSJ designs have occasionally been used in the literature on relative timing, their results have not been interpreted using formal observer models, something which we undertake here.

A note on our terminology seems appropriate at this point. The task we discuss here might reasonably be described as a two-alternative forced choice SJ task. However, taken literally, many tasks can be considered “two-alternative forced choice,” and indeed this description is sometimes applied to SJs and TOJs. Strictly speaking, in the tradition of signal detection theory, 2AFC has additional connotations. Specifically, it implies the presentation of two different exemplars on each trial, between which an observer must discriminate. Hence, in the context of temporal perception, a 2AFC simultaneity judgment would typically involve the presentation of one simultaneous pair of stimuli, and one non-simultaneous pair, in a random sequential order (sometimes referred to as a 2IFC; two interval forced choice) with the requirement to select the synchronous (or, alternatively, the asynchronous) pair. However, the 2AFC designation is inherently ambiguous (regarding whether there are two presentations, or two possible choices) and has not always been used in a manner consistent with the SDT tradition. For this reason, we adopt the clearer “dual-presentation” terminology here.

**Figure 2:**
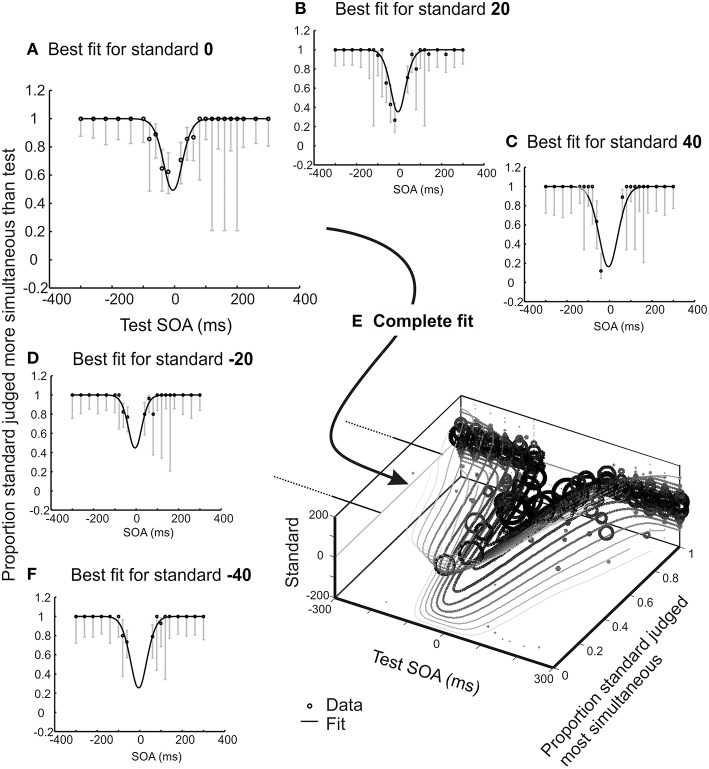
**Illustrative results for one experienced psychophysical observer who completed 15 blocks (2280 trials) of an audio-visual 2xSJ task**. One SOA was selected from a wide range using the method of constant stimuli, the second from a range centered closer to synchrony, using an adaptive method for stimulus selection (providing the roving element; see methods for full details). In order to aid visualization, trials were extracted sequentially in sets associated with a particular standard. In this figure, data and model predictions are averaged for the two possible presentation orders, so the uninformative data points where standard and test are identical have been removed. **(A–D,F)** Observer model fit alongside several data subsets consisting of trials associated with standards ranging from −40 to +40 ms. Error bars show 95% Wilson score binomial confidence intervals, and provide an indication of the number of trials at each stimulus level. **(E)** All data displayed together with the overall model fit. Size of data points correlates with the number of contributing trials.

What observer model might apply to this task? Under the simplest account (c.f. Baron, 1969) each presentation of a pair of stimuli offset by a fixed physical temporal extent would generate a subjective difference in arrival times, and these subjective differences would be variable across successive trials, generating a noisy Gaussian distribution of subjective arrival times, Δ*t*. With two such pairs forming a trial, the observer's task is to compare the absolute subjective differences associated with each pair, to determine which is most simultaneous. Hence the decision variable is the difference in (unsigned) differences in subjective arrival time. To this value a criterion is applied (zero for an unbiased observer) and the observer concludes that the first or second pair is most simultaneous, depending on whether the decision variable falls above or below this criterion. This model and decision process is a special case of one described by García-Pérez and Peli (2014), but is applied here to the temporal rather than the spatial domain. If one pair is always simultaneous (the *standard*) and the other is varied in SOA (the *test*), and order is randomized, the psychometric function (plotting proportion of trials wherein the standard is judged as most synchronous, or equivalently where the test is judged more asynchronous, against the test SOA) is U shaped, with a minimum at the point of subjective simultaneity (see Figure 2A).

In the course of generating data via the 2xSJ described to this point, the experimenter must present a synchronous target on every trial. Unfortunately this seems perfectly designed to train the observer to recognize what a truly synchronous relationship feels like, which is not helpful when we are seeking their natural (potentially non-zero) PSS, or assessing whether this varies with some experimental manipulation. Fortunately a fairly straightforward solution to this problem exists: As experimenters, we should not exclusively use synchronous standards. If neither pair is guaranteed to be synchronous, it becomes difficult for a participant to learn what synchrony is, independent from the perception of synchrony.

**Figure 3:**
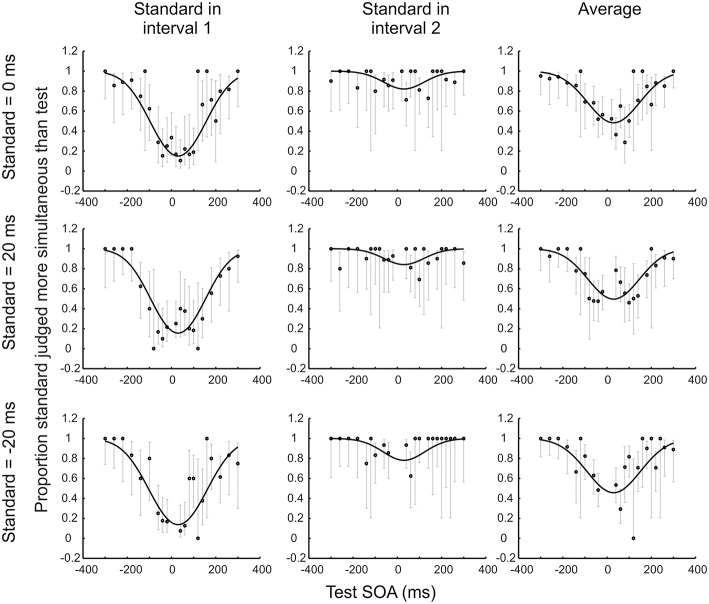
**Illustrative results for one novice psychophysical observer who completed 15 blocks (2280 trials) of an audio-visual 2xSJ task**. Observer model fits are shown alongside several data subsets, consisting of trials associated with standards ranging from −20 to +20 ms. Data are plotted separately based on the order of stimulus presentation (and also in averaged format) across the columns of the figure, to show how an interval bias is captured by the model. For example, imagine that the participant is biased so that, given the question “which was most simultaneous,” they will only pick interval 1 if its duration is at least 30 ms less than that of interval 2 (whereas an unbiased observer would pick interval 1 if its duration was shorter by any margin). If the standard is in interval 1, the chance of picking the standard as more simultaneous is decreased relative to an unbiased observer. By contrast, if the standard is in interval 2, the chance of picking the standard as more simultaneous is increased relative to the unbiased observer. Averaging data from the two possible interval orders simplifies presentation, but the underlying model that is fit to data should ideally still include a parameter to capture any interval bias, because assuming no bias in the presence of an actual bias will lead to an overestimate of sensory noise (although the PSS will still be recovered adequately).

At first glance this procedure seems wasteful, as trials without a synchronous standard do not contribute to the psychometric function shown in Figure 2A. Must they be discarded? The answer is no. The observer model also makes predictions about how often a −20 ms SOA standard should appear more synchronous than a 60 ms SOA test, or any other combination. The only complication is that we must now move from a single SOA vs. proportion correct function to a set of functions, one for each standard (see Figures 2A–F). However, just as with a synchronous standard, each predicted function retains a minimum at the PSS (because a test presented exactly at the PSS will always be more likely than any other test value to be judged as most synchronous, regardless of what value it is being compared to). When the standard is zero, the slopes of the psychometric function are determined largely by sensory noise. This too remains the case for functions predicted for non-zero standards (note the parallel slopes in Figure 2E). In fact, an observer model with just three parameters (one for PSS, one for sensory noise, and one capturing any preference to favor the first pair over the second or vice versa when reporting greater synchrony; see Figure 3 for further explanation of this interval bias) predicts an entire family of psychometric functions. These functions vary in a yoked manner as the model's parameters are adjusted, so best-fitting parameters can be obtained by fitting data from all standards/tests at once.

In the wider literature, “roving” dual-presentation designs like this are sometimes presented as a means to minimize the influence of non-sensory biases, while still measuring a perceptual quality (e.g., Morgan et al., 2013). In brief, these tasks allow the experimenter to apply a contextual manipulation in *both* presentations of a trial, making it less plausible that the manipulation will directly bias the judgment (for example, by nudging a decision criterion in one or other direction). Revisiting the prior-entry example, if an observer must attend the same modality on both presentations, no simple rule such as “pick the stimulus I am attending to” presents itself. However, it is generally possible to conceive of more complex biasing strategies. For reasons of concision, we do not make bias minimization a major focus of our discussion here.

## The present experiments

Our goal here is to demonstrate the use of the roving 2xSJ task as a measure of the PSS. We initially present data from two observers who engaged in a substantial number of audiovisual trials using this task. We use their data to illustrate the fitting procedure (Figures 2, 3), and to evaluate whether simple observer models are plausible. We then present two sets of data each collected from 24 participants, with a much smaller number of trials per participant (which is more representative of typical timing experiments). We additionally collect data in several other tasks, to test whether PSS estimates from different procedures are comparable, and assess correlations across subjects for derived parameters representing both bias (e.g., PSS) and (inverse) precision (i.e., the standard deviation of inferred latency distributions). In Experiment 2, we extend these analyses to judgments involving different combinations of visual, auditory and tactile stimuli. Finally, in Experiment 3, we attempt to replicate a classic effect in the relative-timing literature—endogenous prior entry, using both 2xSJ and TOJ tasks.

## Experiments 1A, 1B, and 1C

### Methods

#### Participants

Participants were recruited (and provided informed consent) according to procedures approved by the City University London Department of Psychology Ethics Committee.

There were two participants in Experiment 1a, one male author (KY) and one female author (SM), initially aged 38 and 24 respectively. Observer KY was highly experienced with relative timing tasks, while observer SM had relatively limited psychophysical experience.

An opportunity sample of 27 naive participants was tested in Experiment 1b. Of these, three were excluded from analysis (see Data Analysis, below) to yield a sample size of 24 (mean age = 24.3, range 18–51, six male). Another opportunity sample of 24 naïve participants was tested in Experiment 1c (mean age = 29.8, range 19–52, 11 male).

#### Apparatus and stimuli

A PC connected to a 20-inch CRT monitor was interfaced with one or more National Instruments A/D cards (DAQCard-6715; DAQPad-6015; X-series PCIe-6323) via a bespoke visual c++ program in order to generate signals and (for the RT task) record responses. Signals (beeps and flashes) were generated at 44100 Hz, and were 10 ms long, with onset and offset slightly smoothed using a Hanning window across the first and last millisecond of the stimulus. The red visual LED signal was otherwise continuous (~60 mcd point source) while the sound was a 1000 Hz sine wave. The LED was placed immediately in front of the center of the monitor, at a distance of ~57 cm from the eyes, so the light subtended a visual angle of ~0.5°. Beeps were presented from a speaker located immediately to the left of the monitor (~30° from the LED) at a comfortable suprathreshold intensity. Responses were recorded via keyboard (for the temporal judgment tasks) or a digital button (for the RT task). Participants fixated the LED during stimulus presentation.

#### Design and procedure

In Experiment 1a both participants completed a 2xSJ task, followed by a TOJ task, followed by a combined SJ and 2xSJ task, with each task typically completed across several days. There was a substantial separation between completing the tasks. In Experiment 1b participants completed three tasks (2xSJ, TOJ, and simple RT) in a single session, with task order counterbalanced across participants. In Experiment 1c participants completed a combined SJ and 2xSJ task. Participants completed 15–20 practice trials before each task, but received no feedback about the correctness of their responses at any time to avoid biasing subjective timing judgments.

In the 2xSJ task, participants in Experiment 1a each completed 15 blocks of 152 trials (i.e., 2280 trials in total), while those in Experiment 1b and 1c completed a single block of 152 trials. There were two flash-beep pairs, and thus two SOA values on each trial. One of the two pairs was selected at random via the method of constant stimuli from the following 19 SOAs (where positive = beep follows flash): −300, −260, −220, −180, −140, −100, −60, −40, −20, 0, 20, 40, 60, 100, 140, 180, 220, 260, 300 ms. Each SOA was selected and presented four times in the first interval, and four times in the second, for a total of 152 trials. The SOA for the other flash-beep pair was selected at random using an adaptive method, such that it would generally be near the PSS, but not always zero (so participants could not infer/learn true synchrony across the experiment). To achieve this, the SOA was drawn from a discrete probability distribution with steps of 20 ms. The initial shape of the distribution was uniform, spanning −60 to +60 ms. However, the distribution had the potential to include values from −300 to +300 ms, and it was updated after each trial based on which of the two presented asynchronies had been selected as more simultaneous. Specifically, the distribution was adjusted so that selection likelihood was increased for all asynchronies ± 40 ms from the asynchrony selected as most simultaneous on that trial. This approach is loosely based on the generalized Pólya urn model (Rosenberger and Grill, 1997) proposed for efficient sampling for temporal order judgments.

In summary—the 2xSJ task involved participants being presented with two flash-beep pairs, with neither SOA being predictable. The pairs were separated by a uniform random 1000–2000 ms interval and participants were required to respond to the question “Which pair was more simultaneous?” using arrow keys on the keyboard. This triggered the next stimulus presentation after 1000–2000 ms. Participants were also given the option to cancel a trial due to inattention, in which case it was repeated at the end of the block.

In the combined 2xSJ and SJ task, stimulus selection and presentation was identical to the 2xSJ task with the following exceptions. Participants were required to make a simultaneity judgment after each stimulus pair, followed by a most simultaneous judgment after every two stimulus pairs. In Experiment 1a, observers completed eight blocks (1216 2xSJ judgments and 2432 SJ judgments). In Experiment 1c, they completed a single block (152 2xSJs and 304 SJs). Each response triggered the next stimulus presentation after 1000–1400 ms, except for the second SJ response, which was followed by the 2xSJ question after 500 ms.

In the TOJ task, for Experiment 1a participants completed 23 blocks of 100 trials each (2300 trials in total). SOA values were selected at random on each trial from an adaptive probability distribution. This distribution was uniform at the start of each block, containing SOAs from −225 to +225 ms in 5 ms increments, but was updated after each accepted trial according to the generalized Pólya urn model (Rosenberger and Grill, 1997; *k* = 32) which attempts to generate test values that sample the full psychometric function efficiently. Distributions could expand to include SOAs from −450 to +450 ms. For Experiment 1b participants completed a single block of 100 trials. In this case the adaptive distribution initially contained SOAs from −140 to +140 ms in 20 ms increments, and could expand to include values from −300 to +300 ms via the generalized Pólya urn method (*k* = 8). In both experiments, after each presentation, participants responded to the question “Which came first (beep or flash)?” using arrow keys on the keyboard. They also had an option to cancel and repeat the trial later. The midpoint of the next flash-beep pair came 1000–2000 ms after each response.

In the RT task (Experiment 1b only) each trial consisted of either a flash or beep, with 50 trials of each type intermixed in random order within a block of 100 trials. Participants responded to each stimulus as quickly as possible using a digital button, following a 1000–2000 ms uniform random response-stimulus interval.

#### Data analysis

For all temporal judgment tasks (2xSJ, SJ, and TOJ) Matlab (The MathWorks Inc.) was used to find maximum-likelihood fits to data (assuming binomially distributed data) with both a null (guessing) model and also a simple independent-channels observer model. The Nelder-Mead simplex algorithm was used to find the best fit. To avoid problems with local maxima, simplex searches were initiated from the factorial combination of several positions per parameter (i.e., a grid search seeded a set of simplex searches). Observer models incorporated a fixed 1% keyboard error/lapse rate, to model occasional errors without increasing parametric complexity (and also simplify the calculation of log likelihood).

Trial-by-trial data for the 2xSJ task consisted of pairs of SOAs, plus a judgment about which pair was most simultaneous. For all trials, the SOA nearest 0 was defined as the standard, as this designation will facilitate a compact presentation of data. All trials where the standard was 0 (i.e., simultaneous) were extracted first, divided according to whether the standard was in the first or the second interval. Remaining trials were then examined, extracting all cases where the standard had an SOA of −20. This was repeated, looking for standards of +20, then −40, then +40, and so on until all trials with at least one SOA of less than ± 200 ms had been extracted. For each standard SOA occurring in each interval, data were plotted to show the proportion of times that the standard was judged as *more simultaneous* than the test (see Figure 2 for examples following averaging across the two presentation intervals, and Figure 3 for examples separated by presentation interval). These functions would be expected to have a minimum near the point of subjective simultaneity.

The tested observer model assumes each stimulus is accompanied by Gaussian noise that will affect its central arrival latency, and that the two stimuli comprising an AV pair may be delayed by neural processing to different extents (generating a non-zero PSS). For each stimulus pair in the 2xSJ, the noisy and delayed signals therefore arrive centrally with latency differences (Δ*t*) that form a Normal distribution of internal responses for any physical SOA:

(1) $$\Delta t_{standard}\sim N\left(SOA_{standard}\;+\;\mu ,\sigma^{2}\right)$$

(2) $$\Delta t_{test}\sim N\left(SOA_{test}\;+\;\mu ,\sigma^{2}\right)$$

Where *SOA*_*standard*_ is the standard SOA (i.e., the stimulus pair that is closest to synchrony), *SOA*_*test*_ is the test SOA (i.e., the other stimulus pair), μ captures any asynchrony specific to the observer (i.e., the PSS) and σ^2^ is the variance contributed by each Δ*t* distribution.

For the subsequent decision, the model assumes that Δ*t* in each pair is converted to an absolute score and that the larger absolute score is judged as less simultaneous. The probability of selecting the standard is therefore:

(3) $$\begin{array}{l} \text{Pr}\left(“\text{Standard}”|SOA_{s\tan dard},SOA_{test}\right)\\ \;=\mathrm{Pr}\left(\left|\Delta t_{s\tan dard}\right|<\left|\Delta t_{test}\right|\right) \end{array}$$

Which, can be written:

(4) $$\mathrm{Pr}\;\left(“\text{Standard}”|SOA_{standard},SOA_{test}\right)=\mathrm{Pr}\left(\frac{\Delta t_{standard}^{2}}{\Delta t_{test}^{2}}<\text{1}\right)$$

Note that $\frac{△t_{standard}^{2}}{△t_{test}^{2}}$ is a random variable with a doubly non-central *F*-distribution. Its numerator's non-centrality parameter is (μ + *SOA*_*t*_)^2^/σ^2^, its denominator's non-centrality parameter is (μ + *SOA*_*n*_)^2^/σ^2^, and both numerator and denominator have one degree of freedom (Morgan et al., 2015). In our Matlab code (available at http://www.hexicon.co.uk/Kielan/) we made use of a saddle-point approximation to the doubly non-central F cumulative distribution function (Butler and Paolella, 2002; Paolella, 2007).

So far, the model simulates an unbiased observer, in the sense of having no preference for the first interval over the second or vice versa. However, Equation (4) can be modified to incorporate an interval bias. Let < *SOA*_*standard*_, *SOA*_*test*_> denote that the standard was presented first and let < *SOA*_*test*_, *SOA*_*standard*_> denote that the test was presented first. Then:

(5) $$\begin{array}{l} \mathrm{Pr}\left(“\text{Standard}”|\;<SOA_{standard},SOA_{test}>\right)\\ \;=\mathrm{Pr}\left(\frac{\Delta {\text{t}}_{standard}^{2}}{\Delta {\text{t}}_{test}^{2}}<\beta \right) \end{array}$$

(6) $$\begin{array}{l} \mathrm{Pr}\left(“\text{Standard}”|\;<SOA_{test},SOA_{standard}>\right)\\ \;=\mathrm{Pr}\left(\frac{\Delta t_{standard}^{2}}{\Delta t_{test}^{2}}<\frac{1}{\beta }\right) \end{array}$$

Under this scheme, the interval bias is proportional, capturing a decision rule in which the observer selects interval 1 (I_1_) when | I_1_| < *c*| I_2_|, and *c* equals β^1∕2^. In words, the biased observer is selecting interval 1 when its duration is less than, e.g., one and a half times the duration of interval 2. This bias can be contrasted with the *constant* bias modeled by García-Pérez and Peli (2014) and presented in their Equations (5), (A5), and (A6) (pages 1676 and 1692). Under this scheme, the observer selects interval 1 when | I_1_|−| I_2_| < *c*. In words, the biased observer is selecting interval 1 when its duration exceeds that of interval 2 by less than, e.g., 50 ms. As we had no a priori reason to favor one form of interval bias over the other, we implemented fits using both, and retained the best fit using either model for each participant in our results.

Recent work by Patten and Clifford (2015) allowed us to derive a closed-form expression for the constant-bias model.^2^ Although our fits were obtained using the (slower to evaluate) derivations described above, we include the new derivation here for completeness, as it is now the default option in our Matlab code:

(7) $$\begin{array}{l} \;\mathrm{Pr}\;\left(“Standard”|\;<SOA_{standard},SOA_{test}>\right)=\\ \{\begin{array}{c} \frac{1}{4}\left(\begin{array}{c} 2-\text{erf}\left(\frac{\Delta t_{standard}-\Delta t_{test}+c}{2\sigma }\right)\left(\text{erf}\left(\frac{2\mu +\Delta t_{standard}+\Delta t_{test}-c}{2\sigma }\right)-1\right)-\\ \text{erf}\left(\frac{2\mu +\Delta t_{standard}+\Delta t_{test}-c}{2\sigma }\right)+\text{erf}\left(\frac{2\mu +\Delta t_{standard}+\Delta t_{test}+c}{2\sigma }\right)+\\ \text{erf}\left(\frac{-\Delta t_{standard}+\Delta t_{test}+c}{2\sigma }\right)\left(\text{erf}\left(\frac{2\mu +\Delta t_{standard}+\Delta t_{test}+c}{2\sigma }\right)+1\right) \end{array}\right),\text{ if }c\leq 0\\ \frac{1}{4}\left(\begin{array}{c} 4+\left(1-\text{erf}\left(\frac{-\Delta t_{standard}+\Delta t_{test}+c}{2\sigma }\right)\right)\left(\left(1-\text{erf}\left(\frac{2\mu +\Delta t_{standard}+\Delta t_{test}-c}{2\sigma }\right)\right)-2\right)-\\ \left(1-\text{erf}\left(\frac{\Delta t_{standard}-\Delta t_{test}+c}{2\sigma }\right)\right)\left(1-\text{erf}\left(\frac{2\mu +\Delta t_{standard}+\Delta t_{test}+c}{2\sigma }\right)\right) \end{array}\right),\text{if }c>0 \end{array} \end{array}$$

Where erf denotes the error function:

(8) $$\text{erf}\left(x\right)=\frac{2}{\sqrt{\pi }}\int_{0}^{x}e^{-t^{2}}dt$$

For our null model, we assumed participants might simply guess, but be biased to choose one or the other interval more often (a one-parameter model). This would lead to deviations from a 0.5 prediction at all test stimulus levels, depending on the interval in which the standard was presented.

To test participant compliance (for exclusion purposes) and the appropriateness of our observer model, we considered two metrics based on deviance of model fit (defined here as −2 × the shortfall in log-likelihood relative to a saturated model). We first assessed whether the more complex (i.e., higher-parameter) observer model provided a significantly better fit than the guessing model. Asymptotically, for nested models the improvement in deviance expected by chance approximates a chi-squared distribution with d.f. equalling the difference in model parameters. We used this result to assess whether the more complex model provided a significantly better fit than its less complex counterpart (at one-tailed *p* < 0.05). If not, there was little evidence that the participant was not simply guessing. No participants in Experiment 1 needed to be replaced on this basis.

For Experiment 1a, we also considered whether our observer model represented a reasonable approximation of the complete psychophysical process. For this purpose we turned to Monte-Carlo simulation. We fed the stimulus values each participant received across the entire experiment into the best-fitting observer model to generate a simulated set of responses. These were then maximum-likelihood fitted with the model, in order to establish a deviance score for the best-fitting model when that model had in fact generated the data. We repeated this operation 1000 times to create a distribution of expected deviances if the model were correct. Finally, we compared the deviance of the model when fitted to *real* data against the simulated distribution of expected deviances, to assess whether the model could be rejected as a full characterization of what observers were doing (two-tailed *p* < 0.05; c.f. Wichmann and Hill, 2001).

For the TOJ task, the same basic observer model assumptions (i.e., Gaussian latency noise) along with the simplest conceivable decision rule (i.e., select order A when Δ*t* is below a decision criterion, otherwise select order B)^3^ predict a cumulative Gaussian psychometric function, where μ is the PSS and σ is the standard deviation of the Δ*t* distribution. The corresponding guessing model has only a single free parameter (a bias for one order over the other, predicting a horizontal line crossing the y axis somewhere between 0 and 1) and is nested relative to the observer model. Hence we assessed whether the observer model provided a significantly better fit than the guessing model (at one-tailed *p* < 0.05) by comparing the change in deviance to a chi-squared distribution with one degree of freedom. In Experiment 1b, three participants were rejected because their performance did not provide evidence to reject the guessing model (i.e., performance was not significantly different from chance). For Experiment 1a we also assessed whether the observer model represented a reasonable approximation of the complete psychophysical process, using the resampling method described above for the 2xSJ task.

For the SJ task, the observer must partition the decision space in a slightly more complex manner than for the TOJ, using two decision criteria in order to report simultaneity only when Δ*t* falls between them. This decision rule predicts a psychometric function that is the difference of two cumulative Gaussians, with their means at the positions of the two decision criteria and their (shared) standard deviation being that of the Δ*t* distribution (Schneider and Bavelier, 2003). To derive a single PSS, a further assumption of some kind is required (for example that the decision boundaries are placed equidistant from subjective zero). Hence we generally prefer to report the two criteria themselves (Experiment 1a) but adopt the equidistance assumption for the purpose of generating a PSS value for correlation analyses (Experiment 1c).

This three-parameter SJ model produces a symmetric psychometric function, but asymmetries are sometimes observed in SJ data (e.g., Yarrow et al., 2011; García-Pérez and Alcalá-Quintana, 2012a,b). If we retain the assumption of Gaussian latency noise, one way to model such an asymmetry is to assume that the two decision criteria might also contribute (independent) noise to the decision (Ulrich, 1987). If the positions of the two decision criteria are considered Gaussian random variables, the resulting psychometric function is the difference of two cumulative Gaussians, but with separate σ parameters (hence a four parameter model; Yarrow et al., 2011).^4^ In our analyses, we fitted both three and four-parameter variants of SJ models. We first asked whether the three-parameter model provided a significantly better fit than a two parameter cumulative Gaussian [deviance improvement, $\chi_{\left(1\right)}^{2}<$ 0.05]. We chose this model in place of a simpler guessing model as it can capture both guessing, and cases where the range of stimuli is sufficient to capture the decision boundary on one, but not both, sides of zero. In Experiment 1c, no participants were excluded on this basis. We then asked whether the four-parameter SJ model provided a significantly better fit than the three-parameter version. If so, we used parameters from the four-parameter fit, taking the lower of the two σ parameters as our measure of precision (as, under this model, it represents an upper bound on the standard deviation of the Δ*t* distribution). This model was used for 7/24 participants.

For simple RT data from Experiment 1b, we first excluded trials with RTs < 100 ms or > mean + (2.5 × SD) ms. The “PSS” was then calculated as the difference between the trimmed mean RT to light and the trimmed mean RT to sound. This gives a measure of the head start sound seems to have relative to light, which can then be compared with the PSS in temporal judgment tasks (Gibbon and Rutschmann, 1969). The starting points for a comparable measure of sensory noise were variances of response times for flashes and beeps in trimmed trials. To generate a measure equivalent to the one obtained in the temporal judgment tasks (i.e., the standard deviation of the Δ*t* distribution) variances for sound RTs and light RTs were summed then square rooted.

For a subset of temporal judgments, we derived bootstrap confidence intervals on best-fitting model parameters. Bootstrap procedures were non-parametric and based on 1999 resamples, using the bias-corrected and accelerated (BCa) method (Efron and Tibshirani, 1994). When considering inferential statistics at the group level, we observed numerous violations of parametric assumptions (e.g., non-normality in difference distributions, Shapiro-Wilks *p* > 0.05). We therefore generally used paired-sample permutation *t*-tests when assessing differences (based on 10,000 permutations) with a *t*_max_ correction for multiple comparisons when three or more conditions were compared. To assess associations, we used the Pearson correlation coefficient, but when there was evidence of non-normality in either of the contributing distributions (Shapiro-Wilks *p* < 0.05) we assessed significance via bootstrap confidence intervals. Unless otherwise noted, we used an alpha value of 0.05 and two-tailed tests.

### Results

**Figure 4:**
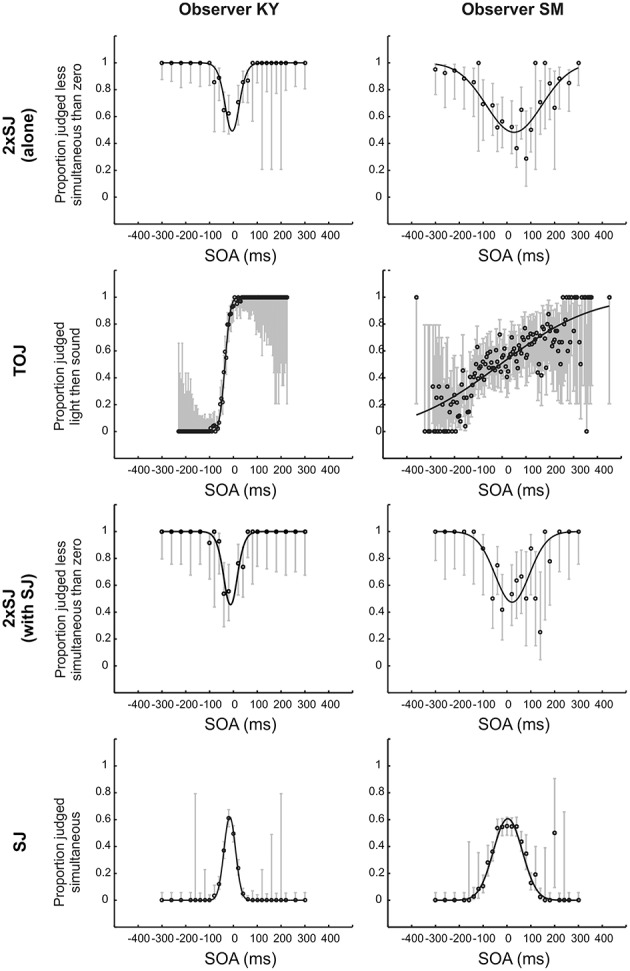
**Fits to temporal judgment data for the two observers in Experiment 1a**. All data are shown for the TOJ and SJ tasks (second and final rows) but just a subset of data (those trials in which one of the two audiovisual stimulus pairs was synchronous, averaged across the two possible presentation orders) is shown for the 2xSJ task (top and third rows). Error bars show 95% Wilson score binomial confidence intervals.

Figure 4 shows raw data for the TOJ and SJ tasks and a subset of the raw data (specifically that with a zero-SOA standard) for the 2xSJ tasks, alongside best-fitting model predictions for both observers in Experiment 1a. By eye, the fits look fairly good. For observer SM, the 2xSJ task simulation suggested that our simple observer model could plausibly be the generating model for the data when the task was performed alongside the SJ, but not when performed alone, as the deviance of the best-fitting model differed significantly from its expected value in the latter case (*p* = 0.048). The observer model described data well for the TOJ task (*p* > 0.05) but not for the SJ task (*p* = 0.03). For observer KY, for the 2xSJ task deviance of the best-fitting model was significantly greater than predicted if our simple observer model were a complete generating model, both when the task was performed alone and alongside the SJ (*ps* < 0.001). However, for the TOJ and SJ tasks, the observer model was plausible (*p* > 0.05).

Parameters derived from these fits are presented in Table 1. PSS values were close to zero for both observers, while latency noise was considerably lower for experienced participant KY than for the more novice participant SM. For SM, noise was much lower in the 2xSJ task than in the TOJ task, despite the model-based equivalence of the two measures (which both correspond to the standard deviation of the difference in arrival times for auditory and visual signals, σ). Confidence intervals indicate this is unlikely to be a chance result. However, noise was very similar between SJ and 2xSJ tasks. It seems SM exhibited a learning effect, as noise was lower for her second run on the 2xSJ task despite the additional requirements of the concurrent SJ task. PSS, however, was similar on both runs. The PSS from the 2xSJ was somewhat higher than that derived from the TOJ, and also than the mid-point of the two boundaries in the SJ (which was 3 ms).

**Table 1:** **Results of TOJ, 2xSJ, and SJ tasks from Experiment 1a, showing estimated model parameters such as point of subjective simultaneity (PSS) and Precision (latency noise; σ) with accompanying 95% confidence intervals (CI) for each observer**.

**Observer**	**Task**	**Parameters**								
		**PSS (ms)**			**Precision (ms)**			**Interval Bias**		
		**Low CI**	**PSS**	**High CI**	**Low CI**	**σ**	**High CI**	**Low CI**	**Bias**	**High CI**
SM	2xSJ (alone)	20	30	39	68	78	86	56	64^a^	78
KY		−8	−5	−2	21	24	27	1.57	1.79^b^	2.05
SM	TOJ	−23	−8	9	273	307	360			
KY		−37	−35	−33	17	19	22			
SM	2xSJ (with SJ)	9	22	22	41	55	55	2	14^a^	14
KY		−15	−12	−9	19	22	26	1.51	1.85^b^	2.27
		**Lower boundary (ms)**			**Precision (ms)**			**Upper boundary (ms)**		
		**Low CI**	**B_*Low*_**	**High CI**	**Low CI**	**σ**	**High CI**	**Low CI**	**B_*High*_**	**High CI**
SM	SJ	−48	−42	−37	50	54	58	44	49	55
KY		−37	−34	−32	20	21	24	1	3	6

a *Best fitting interval bias is constant*.

b *Best fitting interval bias is proportional*.

For experienced observer KY, noise estimates were very similar in all tasks. As for SM, the PSS from the 2xSJ was somewhat higher than that derived from the TOJ task, and also than the mid-point of the two boundaries obtained in the SJ (which was −16 ms). For both observers, confidence intervals were non-overlapping for PSS estimates from the TOJ and 2xSJ tasks, with a more positive PSS in the 2xSJ task. The pattern was similar but slightly less clear cut for the PSS implied by the midpoint of the two decision boundaries in the SJ task. Widths of confidence intervals imply that the PSSs (and boundary estimates for the SJ) were similarly well-estimated by all tasks, whereas the 2xSJ and SJ tasks provided greater confidence regarding true values of latency noise than the TOJ, but specifically for observer SM. Finally, interval bias parameters in the 2xSJ task suggest that both observers showed a bias to favor the second interval.

**Figure 5:**
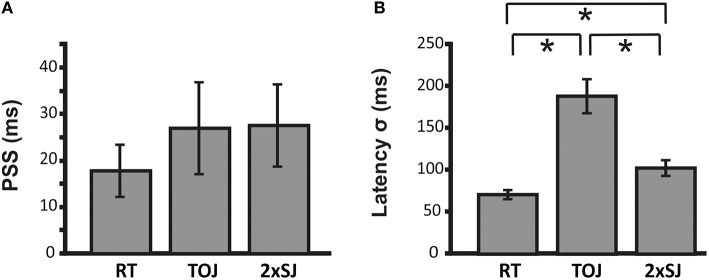
**Mean parameter estimates for PSS and σ for the 24 participants in Experiment 1b**. **(A)** Mean PSS estimates. **(B)** Mean latency noise estimates. Error bars denote standard error of the mean. Asterisks (^*^) denote statistically significant differences (*p* < 0.05).

Moving to the group results from Experiment 1b, Figure 5 shows mean parameters derived from individual fits to data for the 24 participants who successfully completed the experiment. Average PSS estimates were similar for both temporal judgment tasks (TOJ and 2xSJ) and for simple RTs (all pairwise comparison *ps* > 0.05) and in all cases were near zero but slightly positive (i.e., auditory RT < visual RT and simultaneity perceived when audition trails vision), a fairly common finding in audiovisual timing (van Eijk et al., 2008). By contrast, average estimates of latency noise differed significantly across the three tasks [RT vs. TOJ, *t*_(23)_ = 6.35, *p* < 0.001; RT vs. 2xSJ, *t*_(23)_ = 3.49, *p* = 0.005; 2xSJ vs. TOJ, *t*_(23)_ = 4.79, *p* < 0.001]. Noise was highest in the TOJ task, lower in the 2xSJ task, and lowest in the RT task. For the 2xSJ task, 13/24 participants showed a bias to favor the second interval.

We also looked at the mean width of the 95% confidence intervals around estimates derived from the 2xSJ and TOJ tasks. Given that the 2xSJ task included more trials than the TOJ task in Experiment 1b, and would therefore be expected to provide tighter confidence intervals, for this comparison we looked at fits based only on the first 100 trials of the 2xSJ (which still gave mean estimates very similar to those shown in Figure 5, which were based on all 152 trials). For the PSS, confidence limits around estimates were similar for the two tasks [mean widths of 106 ms for 2xSJ vs. 132 ms for TOJ, *t*_(23)_ = 0.94, *p* > 0.05], while for latency noise confidence regions were significantly tighter regarding the lower estimates produced by the 2xSJ task (106 ms for 2xSJ vs. 380 ms for TOJ, *t*_(23)_ = 2.39, *p* < 0.001).

**Figure 6:**
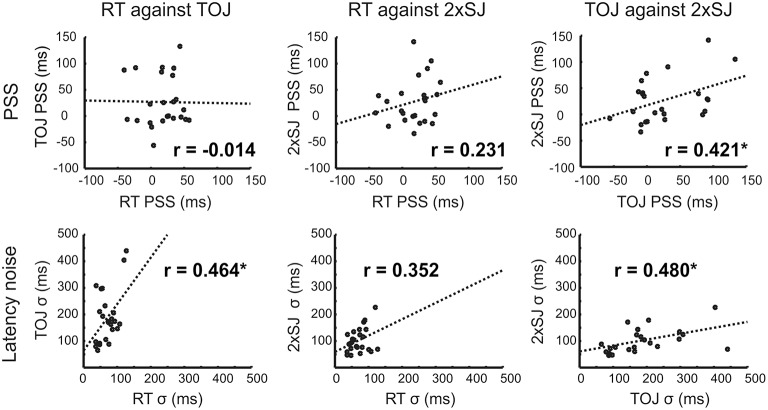
**Scatter plots for correlations in parameter estimates across the 24 participants in Experiment 1b, along with lines of best fit**. Asterisks (^*^) denote significance (*p* < 0.05; PSS, top) or marginal significance (one-tailed *p* < 0.05; σ, bottom).

Importantly, Experiment 1b also provided the opportunity to see whether tasks agreed regarding individual differences in bias (PSS) and precision. Figure 6 shows correlations across participants between equivalent measures obtained with each task. There was a significant correlation between the PSS values estimated from the 2xSJ task and those estimated from the TOJ task (bootstrap *p* < 0.05), but neither correlated significantly with simple RT estimates. For measures of latency noise, correlations between RT and TOJ tasks and between TOJ and 2xSJ tasks were marginally significant (one-tailed bootstrap *p* < 0.05).

**Figure 7:**
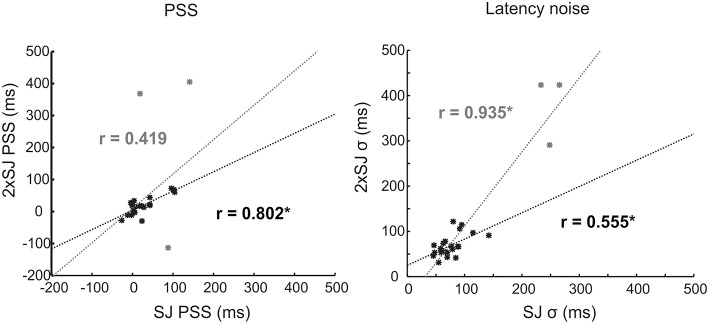
**Scatter plots for correlations in parameter estimates across the 24 participants in Experiment 1c, along with lines of best fit**. Data for outlying participants (and corrlelations/fits for data incorporating those participants) are shown in gray. Data, correlations and fits with outliers excluded are shown in black. Asterisks (^*^) denote significance (*p* < 0.05).

The results of Experiment 1c, where a group of participants made SJs and 2xSJs concurrently, are shown in Figure 7. This illustrates correlations between the two tasks on both PSS and latency noise. Three participants, shown in gray, were clearly outliers in terms of their (in)ability to perform the two tasks, with very high estimates of sensory latency noise. Probably as a consequence of this, their PSS values are also extreme and outlying, suggesting that they have been poorly estimated (note the different axis scales for PSS in Figure 7 compared to Figure 6). We therefore performed correlations both with and without (denoted in gray and black respectively) these outlying participants included. Correlations were significant between tasks on both measures (bootstrap *p* < 0.05) with the exception of the PSS when outlying values were retained.

Concurrent performance of the SJ and 2xSJ tasks yielded mean parameter estimates which did not differ across tasks regardless of whether outlying participants were included in the analysis or not [*with outliers*: mean SJ PSS = 31 ms, mean 2xSJ PSS = 43 ms, *t*_(23)_ = 0.59, *p* > 0.05; mean SJ latency noise = 97 ms, mean 2xSJ latency noise = 108 ms, *t*_(23)_ = 0.91, *p* > 0.05; *without outliers*: mean SJ PSS = 24 ms, mean 2xSJ PSS = 18 ms, *t*_(20)_ = 1.18, *p* > 0.05; mean SJ latency noise = 76 ms, mean 2xSJ latency noise = 69 ms, *t*_(20)_ = 1.37, *p* > 0.05]. For the 2xSJ task, 20/24 participants showed a bias to favor the second interval (binomial *p* < 0.05).

We noticed that, compared to our previous experiences recording SJs on their own, participants appeared to be applying more conservative decision criteria in the SJ task from Experiment 1c. We wondered if the presence of the additional (2xSJ) question was prompting them to be more conservative. As an informal test of this hypothesis, we retrieved a recent data set from 22 participants who completed a baseline SJ task very similar to that used here (but prior to several rather different conditions involving temporal adaptation; Yarrow et al., 2015). Stimuli were virtually identical to those employed here except that the LED flash was green, rather than red. To assess the liberal vs. conservative use of the simultaneous response, we calculated the distance between low and high decision criteria (based on the same four-parameter model fit in both data sets). Data met the assumptions of an independent-samples *t*-test, which revealed that participants placed their decision criteria closer together in the current data set incorporating a concurrent 2xSJ question than in our previous data set with only an SJ question [mean distance with SJ task alone = 440 ms, mean distance with SJ and 2xSJ = 260 ms, *t*_(44)_ = 4.47, *p* < 0.001].

### Discussion

We fitted around 2300 trials from each of two motivated observers and 100–300 trials from each of two sets of 24 typical psychology participants, using simple but plausible models of the TOJ, SJ, and roving 2xSJ tasks. We also recorded simple RTs for one of these groups. Our latency models described the data fairly well for the two observers, but the models were demonstrably incomplete as data were significantly overdispersed. For individual observers, PSS values were more positive when estimated from 2xSJ data than from TOJ and SJ data, but at the group level we found no significant differences between PSS values estimated using our tasks. Group-level estimates of differential latency noise were significantly higher for the 2xSJ task than for the simple RT task, and for the TOJ task than for the 2xSJ task, with the latter result mirrored for our naive observer, but not for our highly experienced observer. Estimates of latency noise were very similar for 2xSJ and SJ tasks when completed concurrently. At the group level, PSS estimates correlated for the TOJ and 2xSJ tasks and for the SJ and 2xSJ tasks, at least when extreme PSS estimates were removed.

The similar and correlated estimates of PSS provided by 2xSJ and TOJ tasks, and by the 2xSJ and SJ tasks, all of which have good face-validity as measures of temporal perception, provide some degree of cross validation for our 2xSJ procedures, and suggest that these tasks are accessing broadly similar cognitive processes. However, the differences in noise parameter estimates, all of which theoretically measure the same quantity (σ), suggest latency variability is not the only source of noise in these tasks, as our modeling naively assumed. The lowest estimate was provided by the simple-RT task, but realistically this must already be an overestimate because the RT task inherits some variability from the motor system that we did not consider formally.^5^ The simple RT task might also rely on sensory pathways somewhat distinct from those used in other timing tasks, but assuming substantial overlap, lower RT noise suggests that the 2xSJ task might gain substantial noise at the decision level, or perhaps as a result of higher memory demands. We can however rule out an interval bias as a possible cause: Although we observed an interval preference, and such biases can have the effect of increasing noise estimates in 2AFC tasks, we explicitly modeled the interval bias for the 2xSJ and thus our estimates are uncontaminated in this respect.

The increase in estimated noise from the 2xSJ to the TOJ task was even more striking than the increase for 2xSJ over the RT task. One possible explanation is that keying errors were more frequent in the TOJ task. It is possible to fit models with additional parameters to describe such errors (Wichmann and Hill, 2001; García-Pérez and Alcalá-Quintana, 2012a,b). However, to do so effectively it is necessary to sample extensively at extreme SOAs where performance asymptotes; for example, fitting SM's data (Figure 4 second panel down on the right) with the lapse rate free to vary actually yielded the same estimate (1%) that had been fixed/assumed in our original fit, and hence also the same estimate of noise. In any case, it is not clear how much explanatory value this kind of account really has, even if it can provide a more appropriate measure of sensory noise, as it still begs the question of why participants are so prone to keying errors in the TOJ task.

The increased noise in the TOJ task might reflect additional processing steps for TOJ over and above those for SJs involving, for example, the binding of event content with event timings (Fujisaki and Nishida, 2005). Another possibility is that values of Δ*t* (i.e., the subjective SOA) near zero cannot be recovered by observers, forcing them to guess in this region (García-Pérez and Alcalá-Quintana, 2012a,b). In this case a lower estimate of sensory noise might be obtained by fitting a TOJ model that explicitly models this low threshold. However, it is worth noting that such operations appear to have had only a limited impact for our more highly experienced observer. For novice participants these operations seem to provide a significantly greater challenge than the extra decision processes inherent in the 2xSJ (which requires that individual SOAs be remembered and compared). The fact that the 2xSJ returns lower estimates of noise is not trivial from a practical perspective, as these values are also better estimated (i.e., sit within tighter confidence intervals) relative to the TOJ. This may make the 2xSJ procedure a more useful task when assessing changes in noise across conditions, although the SJ also appears strong in this regard, and explicit modeling of additional processes might improve estimates for the TOJ.

While there may be some value in employing the 2xSJ in place of the TOJ, the SJ provided similarly low estimates of noise and is clearly a simpler and quicker task to implement. However, we have illustrated how 2xSJ data might be collected at the same time, and our preliminary comparison with previous SJ data (collected without a concurrent 2xSJ task) suggests the additional 2xSJ task encourages participants to use more constrained decision criteria for their SJs. This is potentially valuable, as when participants use very liberal criteria in the SJ, so that many SOAs are judged synchronous almost 100% of the time, any derived PSS value becomes more contentious. Specifically, it will depend to a greater extent on modeling assumptions, for example that participants place their decision criteria at equal distances from subjective time zero. However, our (informal) result would benefit from a more rigorous test, as our data sets differed in respects other than the presence or absence of the concurrent 2xSJ question. Although the set up was broadly similar, LED color, number of trials, and SOA sampling scheme all differed between the data sets we compared.

Having obtained preliminary evidence that the 2xSJ task provides estimates of PSS and latency noise that are broadly compatible with those found using more established tasks, we next determined whether similar correlations could be obtained using stimuli from different modalities (i.e., all combinations of visual, tactile and auditory stimuli) and also with another common temporal judgment task closely related to the TOJ and the SJ, the ternary (SJ3) judgment task.

## Experiment 2

### Methods

Methods in Experiment 2 were identical to those in Experiments 1b with the following exceptions.

#### Participants

An opportunity sample of 6 participants was tested, including two authors (mean age = 27.7, range 20–37, three male).

#### Apparatus and stimuli

Tactile stimuli were vibrotactile sine waves, identical to auditory stimuli except that their frequency was 200 Hz. Vibrotactile stimuli were delivered via a small (~1 cm diameter) ceramic piezoelectric disk coated in plastic. The disk was driven from a custom-built amplifier, and did not produce audible noises with the stimuli we used. It was gripped comfortably between index finger and thumb of the left (non-responding) hand, which rested on participants' laps, around 30 cm from the visual and auditory stimuli.

#### Design and procedure

A 4 × 3 factorial repeated-measures design manipulated both the temporal task (RT, TOJ, 2xSJ, and ternary) and the modality pairing that participants were judging (AV, audiovisual; AT, audiotactile; VT, visuotactile). The four tasks were presented in separate blocks within a single session, always in the same order (ternary, then TOJ, then RT, then 2xSJ). The three modality pairings were completed in separate sessions, with order counterbalanced across participants. For the ternary task, stimulus selection was as per the TOJ task, but in addition to the two order response options, participants could now opt to respond “simultaneous.” If they did so, they were subsequently prompted to take a guess about order (used to update the adaptive distribution from which SOAs were being selected, and discourage excessive use of the simultaneous response option) but these responses were not analyzed.

#### Data analysis

Data in the ternary task were fitted with the same model described previously for simultaneity judgments, except that model predictions were expressed for the three possible response categories, and maximum-likelihood fitting assumed a multinomial data model. To check for sensible responding, goodness of fit was compared against a two-parameter guessing model incorporating guess rates for two out of three response options.

### Results

**Figure 8:**
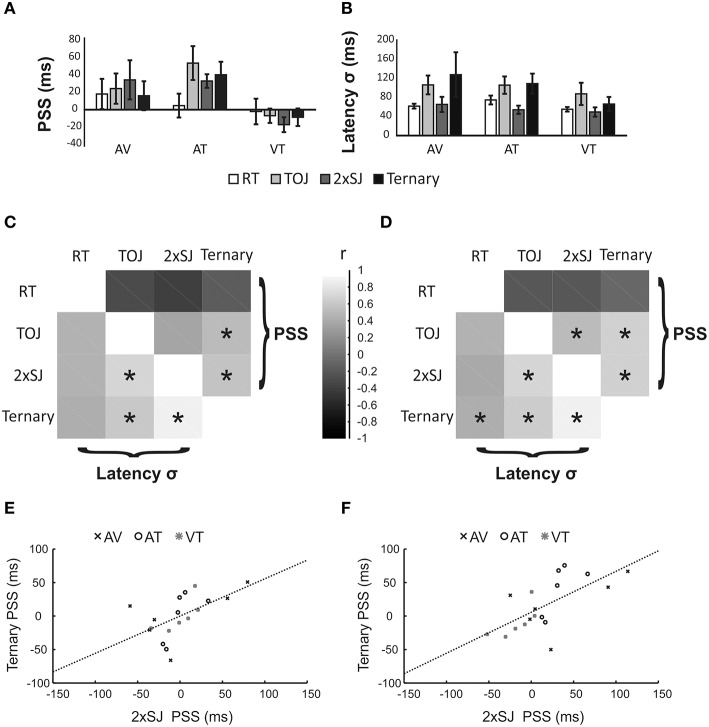
**Results of Experiment 2**. **(A,B)** Mean parameter estimates (PSS and σ) for the six participants in each of four (task) × three (modality pairing) conditions. AV, Audiovisual; AT, audiotactile; VT, visuotactile. Error bars denote standard error of the mean. **(C,D)** Between-task correlations for PSS values (upper right) and σ values (lower left). Data were pooled across all modality pairings, but in **(C)** they were first normalized to the mean value within each modality pairing to remove variance associated with this manipulation. Asterisks (^*^) denote statistically significant differences (*p* < 0.05). **(E,F)** Scatter plots for one illustrative correlation (for the PSS, between 2xSJ and ternary tasks). Data are the same in both plots, but have been normalized (as for **C**, above) in **(E)**.

Figure 8 provides an overview of the results from Experiment 2. Group average PSS and latency noise values are presented in Figures 8A,B respectively. PSS values were once again slightly positive for AV conditions, a trend that was exacerbated for AT conditions but reversed for VT conditions. Different tasks gave quite similar PSS estimates on average (with the possible exception of RT in the AT condition). Estimates of latency noise were similar between modality pairings, but appeared lower in RT and 2xSJ tasks compared to TOJ and ternary tasks. However, no differences between tasks reached statistical significance for either PSS or latency noise (perhaps reflecting the small sample size in this experiment).

In order to increase power to detect correlations, we combined data from all six observers and three modality pairings into 18 points. Differences between pairings might lead to a clustering of data into three sets. Hence any correlation would be driven in part by the common effect of a particular modality pairing on measures from two or more tasks. Although we consider this essentially legitimate (i.e., if a change of modality pairings affects the PSS from two tasks in the same way, this is reasonable evidence that the two tasks are indexing similar mental operations) we also performed correlations after first normalizing data within each modality pairing. We did this by subtracting the mean for that pairing, so that only differences relative to the mean remained to be correlated between tasks.

Correlations are shown if Figures 8C–F. Figures 8C,D summarize correlations between all four tasks for both PSS and latency noise. Broadly, correlations between equivalent measures of latency noise are positive for all task pairs, whereas correlations between measures of PSS are generally low and slightly negative between the RT task and the other tasks, but high and positive between the three temporal judgment tasks. Focussing on the critical correlations between the 2xSJ task and the other tasks (and omitting marginal and non-significant results), with normalization there was a significant PSS correlation between the 2xSJ task and the ternary task (*r* = 0.575, *p* = 0.013), and a significant latency noise correlation between the 2xSJ task and both the ternary task (*r* = 0.881, bootstrap *p* < 0.05) and the TOJ task (*r* = 0.707, *p* = 0.001). Without normalization, correlations were generally slightly higher. Here, there were significant PSS correlations between the 2xSJ task and both the ternary task (*r* = 0.623, *p* = 0.003) and the TOJ task (*r* = 0.527, *p* = 0.025). Similarly, for latency noise there were significant correlations between the 2xSJ task and both the ternary task (*r* = 0.877, bootstrap *p* < 0.05) and the TOJ task (*r* = 0.708, *p* = 0.001). The scatter plot for the correlation between the ternary and 2xSJ tasks is shown in Figure 8 parts E and F (for normalized and non-normalized, data respectively).

### Discussion

In Experiment 2, we had observers make temporal judgments and rapid button presses in response to audiovisual, audiotactile, and visuotactile stimuli. The overall pattern of mean PSS values we recovered using four different tasks was similar across tasks. A simple reading would be that the auditory pathway is somewhat shorter than both the visual and tactile pathways, with the difference being greatest between auditory and tactile pathways. However, this result is likely to be stimulus specific and other interpretations are possible. For our purposes, the more important result is that the 2xSJ task provided results comparable to other temporal judgments tasks, and correlated with them for both PSS and latency noise measures (although as in experiment 1b, correlations with RT were lower for latency noise and absent for PSS). In particular, the new correlation between PSSs obtained using 2xSJ and ternary judgment tasks (based on several modality pairings) corroborates those previously obtained in Experiment 1b and 1c using TOJ and SJ tasks (with only AV stimuli).

Having found further evidence for the utility of the 2xSJ task when assessing a baseline PSS, we wanted to determine if it can also provide sensible estimates of *changes* in PSS across experimental conditions. For this purpose we attempted to recreate a classic experimental effect from the literature—cross-modal prior entry—tested with both 2xSJ and TOJ tasks.

## Experiment 3

### Methods

Methods in Experiment 3 were identical to those in Experiments 1a–c with the following exceptions.

#### Participants

An opportunity sample of 11 naive participants was tested, with three excluded from further analysis as one or both of the observer models failed to fit their data better than the relevant chance model in one or more conditions. This yielded a sample size of 8 (mean age = 33.5, range 18–52, two male).

#### Apparatus and stimuli

Auditory stimuli were delivered through headphones (Sennheiser PX360). In order to manipulate the allocation of attention, a subset of stimuli were modified to become targets in a (secondary) detection task. In contrast to the usual stimulus duration of 10 ms, these stimuli had durations of 17 ms (for auditory targets) or 25 ms (visual targets).

#### Design and procedure

A 2 × 2 factorial repeated-measures design manipulated both the temporal task (TOJ vs. 2xSJ) and the modality that participants had to monitor for targets in an additional detection task (auditory vs. visual). The four conditions were presented in separate blocks, with order counterbalanced across participants in a nested fashion (i.e., four possible orders, where each task could be completed first or second, and nested within that ordering each modality could be attended first or second). In addition to the two response options for temporal judgments (outlined in Experiments 1a and 1b), participants now received a third alternative—to indicate that a target had been present (in which case they were told not to worry about the temporal judgment). Accurate feedback was provided regarding the secondary detection task, flagging hits and misses on target-present trials and false alarms on temporal-judgment trials.

Blocks contained 190 trials, with 80% non-target (i.e., temporal-judgment) trials and 20% target trials. Targets were presented only in the monitored modality. The extra dual-task requirement made the temporal tasks more difficult. To counter this, for the 2xSJ task SOAs ranged more widely. One stimulus was drawn from the following 19 SOAs: −375, −325, −275, −225, −175, −125, −75, −50, −25, 0, 25, 50, 75, 125, 175, 225, 275, 325, 375 ms (with each SOA occurring five times in each interval across a block of trials). The second SOA was drawn from a discrete probability distribution with steps of 25 ms, initially uniform, spanning −75 to +75 ms, but potentially expanding to ±375 ms in an adaptive manner. For the TOJ task, SOA values from −450 to +450 ms were used (in 30 ms steps). This distribution was initially uniform across this entire range except for the two most extreme values, which were nine times more likely to occur than each of the 29 other SOAs (prior to adaptive updating).

### Results

In the secondary task, participants tended to detect targets successfully, but performance was imperfect. Hits and false alarms were converted to d-prime (*d*′) values (Green and Swets, 1966), with average *d*′ for the group ranging from 2.22 for visual-target TOJ trials (79.3% hits, 6.3% false alarms) to 4.44 for auditory-target 2xSJ trials (96.1% hits, 0.3% false alarms). Hence there was an incentive to attend the modality containing detection targets.

**Figure 9:**
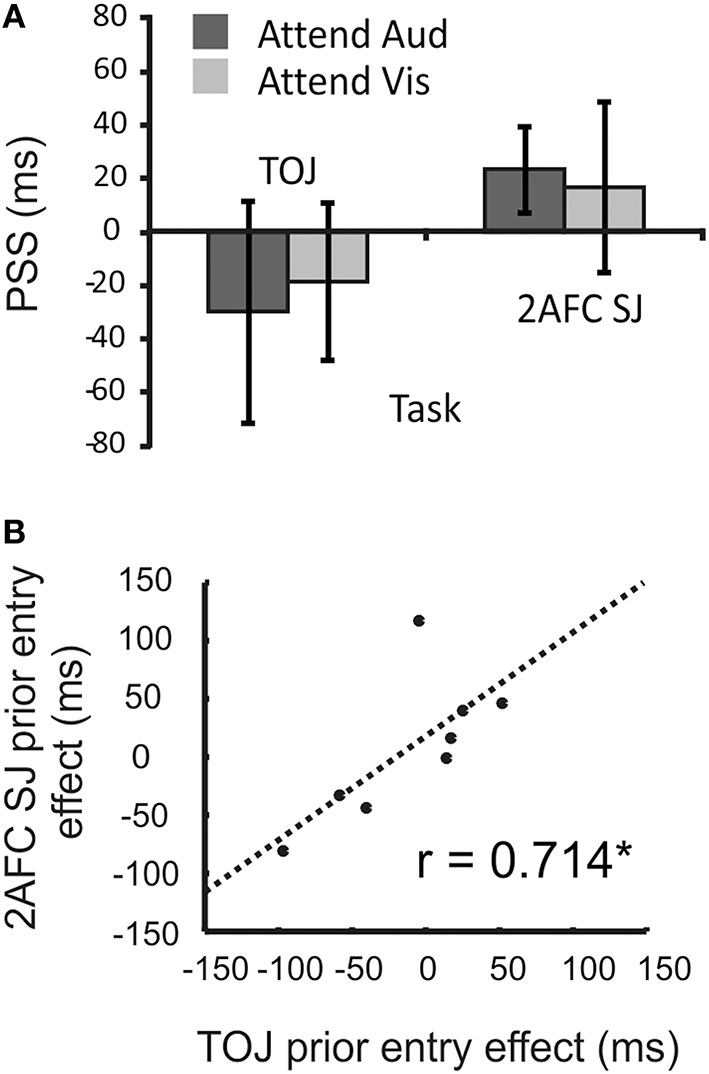
**Results of Experiment 3**. **(A)** Mean PSS when a secondary task promoted attending to audition or vision, estimated using TOJ and 2xSJ tasks. Error bars denote standard error of the mean. **(B)** Scatter plot of correlation in prior-entry effect magnitudes (PSS when attending to audition minus PSS when attending to vision) alongside line of best fit. The asterisk (^*^) denotes a significant correlation (*p* < 0.05).

We expected to see the PSS become more positive when participants attended audition relative to when they attended vision (as the auditory signal should be sped in the brain, and thus require a physical delay to seem synchronous). However, as shown in Figure 9A, on average the target modality (and thus the presumed allocation of attention) had no effect on PSS values estimated via either the TOJ task [*t*_(11)_ = 0.66, *p* > 0.05] or the 2xSJ task [*t*_(11)_ = 0.35, *p* > 0.05]. Furthermore, there was no evidence for a different magnitude of prior-entry effect between TOJ and 2xSJ blocks [with effect magnitude being the difference in PSS between auditory and visual-target conditions; *t*_(11)_ = 1.24, *p* > 0.05]. However, when we examined the prior-entry effect on a participant-by-participant basis, comparing the effect's magnitude derived using the TOJ task with that obtained using the 2xSJ task, a significant correlation emerged (*r* = 0.71, *p* < 0.05; see Figure 9B).

### Discussion

In Experiment 3 we manipulated attention, directing it toward either the visual or auditory modality via a strategic incentive (to maximize performance on a concurrent detection task), while measuring changes in PSS via both a TOJ task and a roving 2xSJ task. We failed to obtain a prior-entry effect on average across participants, but did obtain a significant correlation between attentional influences on our two timing tasks.

It is not uncommon to fail to find cross-modal prior entry, particularly with manipulations of endogenous attention (e.g., Cairney, 1975). We used a 100% predictive instruction (i.e., targets always came only in the attended modality) so cannot offer any independent evidence that attention was allocated as we envisaged, but we think it likely on strategic grounds. Our manipulation of attention could be considered to be either between modalities or between spatial locations or, most likely, between both of these (as target stimuli came from either a fixated LED or via headphones). However, this manipulation had no significant effect on the PSS for our sample. Perhaps there really is no consistent effect to find, or perhaps the average effect is very small (e.g., associated latency changes in ERP components are tiny; Vibell et al., 2007) and we lacked power to demonstrate it.

We did, however, find evidence for a correlation in the (non-uniform) effects of attention on PSS estimates across participants. This correlation is interesting for two reasons. First, it demonstrates that while the experimental manipulation did not have a consistent effect on all participants, it influenced each participant's PSS in an individually reliable fashion (as revealed by the matched effects obtained using two different temporal judgment tasks in separate blocks of trials). Second, it provides further evidence that TOJ and 2xSJ tasks tap similar temporal processes.

## General discussion

In this paper, we (1) considered the merit of a roving 2xSJ task for estimating the bias and precision of temporal judgments; (2) provided predictions for a simple but theoretically-derived observer model, and; (3) benchmarked the task against more established TOJ, SJ, and ternary tasks when estimating both baseline PSS and (for the TOJ) changes in PSS. We found that the 2xSJ task was manageable for typical psychology participants; that the observer model was a somewhat useful approximation (albeit a simplification) of the full psychological process of temporal judgment; and that the 2xSJ task provides estimates of the PSS that are comparable to those obtained using other temporal judgments. The 2xSJ task is, however, likely to provide lower and less variable estimates of sensory noise than the TOJ, at least when the TOJ is modeled without additional cognitive operations such as guessing. On this basis we believe that the 2xSJ task has validity as a supplementary measure of temporal experience. From a practical perspective, we would recommend that researchers primarily consider using it in concert with the classic SJ (i.e., as an additional question) when one of the following two conditions apply:

1. When an estimate of the PSS is desirable between two quite different (e.g., bimodal) stimuli and there are reasons to believe that the SJ task will give rise to “synchronous” reports over a fairly broad range of SOAs (e.g., when stimuli are naturalistic/noisy or participants are not practiced). In this case, the 2xSJ should encourage the use of more conservative criteria regarding simultaneity (benefitting the fitting and interpretation of the SJ data) and provide an additional and less theoretically dependent point estimate of the PSS.
2. When a single-presentation task such as the TOJ or SJ seems likely to encourage decision biases, for example when the experimental manipulation could be seen to suggest one of the two possible answers when the observer is uncertain (e.g., a directive to preferentially attend one of two events).

In these situations, the 2xSJ should result in low and fairly stable estimates of sensory noise alongside a PSS that is less dependent upon the placement of decision criteria. However, such benefits must be weighed against the increased experimental time necessary to complete each trial.

We have considered five tasks here, but our main focus was the 2xSJ task. We obtained a significant correlation between PSS estimates from this task and other temporal judgment tasks, and also between changes in PSS estimates across conditions for the 2xSJ and TOJ. Several previous studies have attempted to find correlations between PSS values estimated via more than one task. For example, both van Eijk et al. (2008) and Love et al. (2013) failed to find any correlation between the PSS estimated from a temporal order judgment and that estimated from a synchrony judgment, while Freeman et al. (2013) found a surprising negative correlation between PSS for audiovisual speech (estimated via TOJ) and the maxima of the function describing the probability of McGurk integration across different SOAs. We suggest previous failures to obtain correlations between TOJ and SJ tasks might reflect the different decision processes in these two tasks. In particular, the SJ is fundamentally a method for obtaining a *region* of subjective simultaneity, rather than a *point* of subjective simultaneity. To infer a PSS, it is necessary to make some assumption about how the two criteria for demarcating synchrony from asynchrony are selected (e.g., that they are placed symmetrically about a subjective Δt value of zero). However, participant strategies might vary, with a concomitant effect on the inferred PSS. Such strategic variability might make correlations difficult to detect. Our 2xSJ task, although still based on a judgment of simultaneity, forces observers to decide which SOA seems *most* synchronous, which might be more comparable to the SOA at which their impression of order switches in TOJs^6^.

Many studies have also reported differences between PSS estimates obtained using TOJ and SJ tasks. For example, Linares and Holcombe (2014) found that for four of seven participants, confidence intervals around the PSS did not overlap for TOJ and SJs. We obtained a similar result for two observers in Experiment 1a when comparing TOJ and 2xSJ parameters, although this difference was not apparent in the group data from Experiment 1b. Interestingly, Linares and Holcombe (2014) also found differences between PSS estimates obtained using a TOJ task and those obtained using a AV-VA (or VA-AV) duration comparison task, which shares a broad structural similarity with our 2xSJ task, but uses just a few rather longer durations. Differences in PSS values obtained using these kinds of tasks might reflect a common decision-level bias in the TOJ (e.g., observers tend toward one response when uncertain). Alternatively (or additionally) there might be an asymmetry in the transducer function that relates objective to subjective time for AV intervals relative to VA intervals (e.g., if AV time accrues more quickly than VA time at a subjective level, perhaps due to differences in arousal or attention). This could bias PSS estimates derived using both 2xSJ and interval-comparison tasks.

We did not obtain correlations between PSS estimates from our temporal judgment tasks and that estimated from simple RT, although there was some evidence for a correlation in estimates of noise involving RTs and other tasks. There is a previous literature examining the extent to which simple RT and TOJ tasks rely on the same sensory representations, with the main focus being the tendency for PSS estimated from TOJs to dissociate from that estimated using simple RT following experimental manipulations such as changes in stimulus intensity (see e.g., Jaskowski, 1999, for a review of the early work). Here too dissociations may be explicable in terms of different decision strategies being applied to different tasks (Miller and Schwarz, 2006; Cardoso-Leite et al., 2007) rather than implying a complete mechanistic separation. Our mean PSS estimates actually matched fairly well between RT and temporal judgments, and the absence of a correlation is perhaps explicable in terms of fairly low experimental power combined with differences between these tasks from the decision level onwards.

Estimates of latency noise were often correlated between our various tasks. While this finding is consistent with a common sensory stage accessed by different tasks, as implied by independent-channels models, it might also have resulted from quite general cognitive factors, such as the ability to maintain focussed attention during a long, boring task. Stevenson and Wallace (2013) have previously reported correlations between measures of a construct known as the temporal binding window, derived using several of the tasks we assess here. They constructed this measure by fitting one or more sigmoids in a piecewise manner to their data, and calculating the difference between threshold values. It is rather difficult to map this kind of measure, which is likely to conflate latency noise and decision criteria (to different extents depending on the exact task) onto the model-based measures we derive here, but our findings are broadly consistent with theirs.

Fitting observer models to data is generally preferable to fitting arbitrary functions, as derived parameters will have clearly defined meanings. However, this is only true to the extent that the models are accurate. The observer models we develop and use here are very simple (too simple in several cases) but seem a reasonable starting point. There are many more complex variants that might be considered, and indeed some such variants have been shown to perform well for TOJ, SJ, and ternary tasks (García-Pérez and Alcalá-Quintana, 2012a,b, 2015). One example of the additional complexity we have omitted is the well-known scalar property (the variant of Weber's law that applies to time) as we have assumed constant noise alongside an affine transformation from objective to subjective SOAs. It remains to be seen whether more complex models that incorporate such features will provide a significantly better fit to temporal judgment data when their additional parametric flexibility is taken into consideration.

## Conclusions

We have outlined methods and analysis procedures for implementing a roving 2xSJ task, useful for determining both a point of subjective simultaneity and associated judgment precision estimates for subjective timing. This task returns PSS estimates that seem largely consistent with those returned by more traditional tasks, but in some cases provides lower and more constrained estimates of sensory noise, perhaps indicative of a more straightforward decision process. It does so while explicitly requiring participants to decide which alternative timing relationship is *most* synchronous on any given trial (rather than revealing what range of relationships are sometimes described as synchronous). It can also easily be combined with judgments about each stimulus. It therefore provides a useful complement to existing methods for investigating subjective timing.

## Author contributions

KY and DA conceived and designed the work. SM and SD acquired the data. JS, KY, SM, and SD analyzed the data. All authors interpreted the data and drafted and approved the manuscript.

### Conflict of interest statement

The authors declare that the research was conducted in the absence of any commercial or financial relationships that could be construed as a potential conflict of interest.

## Footnotes

^1^This assertion clearly needs to be examined case by case. For example, consider the literature on temporal recalibration (Fujisaki et al., 2004; Vroomen et al., 2004). This effect, presumed to be a form of adaptation, is revealed when participants are repeatedly exposed (i.e., adapted) to a non-zero asynchrony between different kinds of event (e.g., beeps that consistently lag after flashes). It this situation their PSS has been shown to change; participants now respond as though the relationship to which they have been adapted appears more synchronous than it seemed prior to the adaptation. However, If we repeatedly expose our participants to a particular asynchrony, it is not difficult to imagine that they might come to form a belief that this relationship is important, biasing their interpretation of any subsequently presented/judged asynchronies when forced to categorize them as simultaneous or not. Note, however, that some recent evidence suggests that temporal recalibration is not entirely the result of a decision-level bias (Roseboom et al., 2015).

^2^Thanks to Kai Schreiber for help with the derivation.

^3^Described as a “deterministic” decision rule by Sternberg and Knoll (1973).

^4^This model occasionally breaks, because differential levels of criterion noise and tight decision criteria imply that the two component cumulative Gaussians overlap. This can be resolved by turning to simulation and requiring that the decision boundaries never take an illogical order, but here we instead simply assigned zero likelihood to fits generating impossible probabilities.

^5^It is, however, possible that we underestimated noise slightly in the RT task, as we relied on data trimming to exclude outliers.

^6^Of course the TOJ faces its own issues as a measure of maximal synchrony perception, not least the fact that it doesn't actually ask about synchrony directly, only about perceived order.
